# Protocol for the evaluation of a free health insurance card scheme for poor pregnant women in Mbeya region in Tanzania: a controlled-before and after study

**DOI:** 10.1186/s12913-015-0905-1

**Published:** 2015-07-04

**Authors:** Josephine Borghi, Kate Ramsey, August Kuwawenaruwa, Jitihada Baraka, Edith Patouillard, Ben Bellows, Peter Binyaruka, Fatuma Manzi

**Affiliations:** Ifakara Health Institute, Kiko Avenue, Dar es Salaam, Tanzania; Department of Global Health and Development, London School of Hygiene & Tropical Medicine, 15-17 Tavistock Place, London, WC1H 9SH UK; Columbia University, Mailman School of Public Health, New York, NY USA; Population Council, Nairobi, Kenya

**Keywords:** Demand-side financing, Health insurance, Maternal health, Poverty, Impact evaluation, Process evaluation, Economic evaluation

## Abstract

**Background:**

The use of demand-side financing mechanisms to increase health service utilisation among target groups and enhance service quality is gaining momentum in many low- and middle-income countries. However, there is limited evidence on the effects of such schemes on equity, financial protection, quality of care, and cost-effectiveness. A scheme providing free health insurance cards to poor pregnant women and their households was first introduced in two regions of Tanzania in 2011 and gradually expanded in 2012.

**Methods:**

A controlled before and after study will examine in one district the effect of the scheme on utilization, quality, and cost of healthcare services accessed by poor pregnant women and their households in Tanzania. Data will be collected 4 months before implementation of the scheme and 17 months after the start of implementation from a survey of 24 health facilities, 288 patients exiting consultations and 1500 households of women who delivered in the previous year in one intervention district (Mbarali). 288 observations of provider-client interactions will also be carried out. The same data will be collected from a comparison district in a nearby region. A process evaluation will ascertain how the scheme is implemented in practice and the level of implementation fidelity and potential moderators. The process evaluation will draw from impact evaluation data and from three rounds of data collection at the national, regional, district, facility and community levels. An economic evaluation will measure the cost-effectiveness of the scheme relative to current practice from a societal perspective.

**Discussion:**

This evaluation will generate evidence on the impact and cost-effectiveness of targeted health insurance for pregnant women in a low income setting, as well as building a better understanding of the implementation process and challenges for programs of this nature.

## Background

Stagnating maternal and neonatal indicators in many countries of Sub-Saharan Africa are a major concern for national governments and development partners striving to achieve the Millennium Development Goals (MDGs) [1, 2]. Universally, these indicators are poorest among low-income populations. A complex combination of supply and demand side factors limits the use of essential maternal and newborn health services holding back improvements in outcomes. There have been many studies examining the determinants of skilled attendance at delivery; however, the emphasis has been on individual and household characteristics more than on supply side factors that may affect demand [3], such as cost and quality of care.

Although maternal and under-five services are officially exempt from user fee payment in public facilities in many countries [4], in practice exemptions are not always consistently implemented [5–7], as health facilities generally do not receive financial compensation for the foregone user fee revenue [8]. The financial incentives of providers to maximize facility revenue are at odds with a policy which would reduce that revenue quite substantially by providing free services to certain groups.

Quality of care can often be very poor, especially in lower level rural public facilities. Evidence suggests that quality is an important determinant in household decisions to seek care, especially for delivery [9].

In recognition of the cost and quality barriers to care seeking, demand side financing strategies have been proposed as a mechanism to channel subsidies to the patient directly ([10–14]) and promote quality of care by requiring minimum quality standards for accreditation. Vouchers are one such demand side financing mechanism [15–17]. Vouchers have been found to increase service utilization and quality among specific population groups [18, 19]. With donor support, a number of countries are now implementing voucher schemes with a view to increasing coverage and improving the quality of reproductive and child health services. A number of evaluations of these schemes are currently underway (e.g. [20, 21]). Evidence so far points to vouchers having a positive effect on utilization of facility-based deliveries and antenatal care [18, 22–25]. However, population awareness levels have been found to be low and implementation challenges when dealing with vulnerable groups and sensitive topics (for example, gender based violence services) have also been documented [26]. There is less evidence available on the effects of vouchers on provider organization, quality of care received by clients and on financial protection and equity [27]. There is very limited evidence of the cost-effectiveness of such schemes [27] with the exception of one study [28].

Similar to vouchers, the provision of subsidized health insurance cards to vulnerable groups would allow recipients to benefit from services covered by insurance without having to pay a premium. The insurance fund would reimburse providers, and could promote quality through accreditation, and be used to expand client service choice. In 2003, the government of Ghana introduced free National Health Insurance Scheme (NHIS) cards for vulnerable groups including pregnant women [29]. Nigeria also has an insurance scheme that provides subsidized insurance for pregnant women; however, population coverage is very limited. Other countries, including, for example, Paraguay and Argentina, have prioritized access to maternal health services through health insurance [30, 31]. There have been many evaluations of the impact of health insurance on service coverage and financial protection, with effects generally being positive (e.g. [32–37]). However, the evidence of the effect of programmes offering free health insurance cards to poor pregnant women in the African region is more limited [29, 38].

### Free health insurance cards for poor pregnant women in Tanzania

The National Health Insurance Fund (NHIF) was set up as a mandatory insurance scheme for the public formal sector in 2001, and now also attracts clients from the private sector. The NHIF offers free outpatient and inpatient care including surgeries with limited exclusions to its members and in 2011 population coverage was estimated at just over seven percent [39]. Drug costs are reimbursed from selected pharmacies. All government health facilities are accredited irrespective of the quality of care they provide, and many private for profit and faith-based (FBO) facilities that meet pre-defined quality standards^1^ are also accredited.

Currently unemployed individuals or those working outside the formal sector are not eligible for NHIF coverage, but can join a community based health insurance scheme which provides access to primary health care with limited referral care for its members, the Community Health Fund (CHF), which is managed by the NHIF; however, enrolment levels remain low (just over 5 % in 2011 [39] of the population).

Care for pregnant women and children under 5 years of age is officially free at public facilities; however, in practice, exemptions for these groups are not systematically implemented [5]. Further, poor households should officially be identified by village leaders and their fees waived in public facilities. However, the lack of clearly defined criteria to identify the poor limits this practice [40].

In 2010 the Tanzania National Health Insurance Fund (NHIF) with technical support from GFA Consulting group, Institute for Health and Social Research (Institut für Gesundheits und Sozialforschung GmbH, IGES) and Mennonite Economic Development Associates (MEDA) began implementing a scheme that consists of providing health insurance to poor pregnant women and their households in Mbeya and Tanga regions. This scheme is funded by the German Development Bank: KfW and is locally referred to as: the Helping Poor Pregnant Women Access Better Health Care Project, hereafter referred to as the ‘KfW scheme’.

The KfW scheme aims to provide free NHIF membership to poor pregnant women during pregnancy and for up to 3 months after delivery. In addition, CHF cards are provided to the woman’s family offering insurance cover for a year from the date of enrolment. It is expected that by exposing households to the CHF, demand for enrolment may be stimulated, increasing national health insurance coverage. Two approaches to targeting the poor are employed. In most districts, all women will be eligible to participate in the scheme (geographical targeting). In more wealthy districts, a score card system of poverty identification developed by the NHIF will be used to identify poor individuals (individual targeting).^2^

The KfW scheme was first implemented in two districts in Mbeya and Tanga regions. After six months of implementation, the scheme was scaled-up to the remaining districts in each region.

This paper presents the protocol for the evaluation of the KfW scheme in one district in Mbeya region, providing an overview of the framework and methods of the evaluation.

### Evaluation framework

**Fig. 1:**
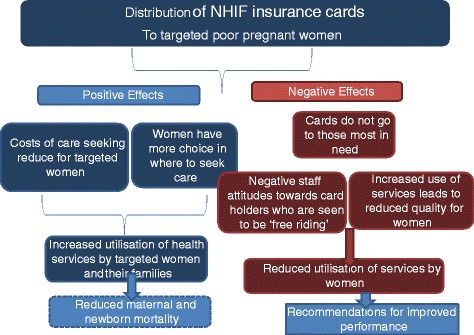
Pathways of change

The KfW scheme is expected to have an impact on maternal and newborn health status through pathways on the demand for and supply of health services. There are also a number of potentially unanticipated consequences (or risks) of such a scheme that need to be monitored, in order to provide timely recommendations for improved implementation. Figure 1 presents a simplified overview of the theory of change underpinning the evaluation that was developed with reference to existing literature and based on discussions within the evaluation team.

The impact of the scheme will depend upon the degree of implementation and the extent to which the scheme is implemented as designed, including the appropriate and effective targeting of women for receipt of the free NHIF card, and the manner of providing reimbursements to health facilities.

It is hypothesised that, if fully implemented, the scheme would significantly increase health service utilisation among targeted women, by removing the financial barriers that poor women face in terms of purchasing health services and drugs, although women would still incur transport costs and may face other access barriers. Furthermore, the women would have a wider range of choice in terms of care seeking. Poor women regularly seek care at lower level public facilities which are low cost but may be of more limited quality [41]. In some areas of Tanzania, women choose to deliver at home because they cannot afford the costs of care that is perceived to be of higher quality [42]. With the KfW scheme they would be able to choose to seek care from accredited faith-based and private for profit providers if desired. The quality of services provided to programme beneficiaries may also be higher if the providers perceive card users to be bringing in more revenue than women under the exemption scheme. Quality may be improved if the reimbursements from the scheme can be re-invested in facilities to reduce stock outs of drugs and medical supplies and undertake minor renovations where needed.

However, in parallel, the scheme may negatively affect provider attitudes towards those without health insurance in public facilities who are supposed to receive free care. Quality of care may also decrease (e.g. greater waiting times, poor provider behaviour) with higher levels of utilisation, unless there are offsetting investments in staff and supplies. If providers are not well informed about the scheme, they may see women with cards to be ‘free riding’ and not appreciate that the facility will be reimbursed for the care provided. Initial increases in utilisation brought about by the scheme may reverse if there is reduced quality of care and negative staff attitudes towards card holders. It is important to document unintended consequences and use this to feedback to implementers to improve performance.

### Objectives of the evaluation

1. To measure the effect of the KfW scheme on the quality, coverage and costs of healthcare services provided to women and their families at health facilities.
2. To monitor the process of implementation including: the acceptability of the KfW scheme to beneficiaries and implementers, the fidelity of implementation, and the context of implementation.
3. To measure the cost-effectiveness of the KfW scheme.

To address these objectives, there are three components to the evaluation: an impact evaluation, a process evaluation, and an economic evaluation. The specific objectives and methods of each component of the study are reviewed in turn.

## Methods

### Impact evaluation

#### Study design

The impact evaluation will employ a controlled before and after study design. Surveys will be undertaken in one district (Mbarali) in Mbeya region before and after the introduction of the KfW scheme and also in one comparison district with no scheme (Kilolo). The comparison district was selected from a neighbouring region and was similar to the intervention district in terms of baseline CHF coverage, poverty and literacy rates, population density and population per health facility.

**Fig. 2:**
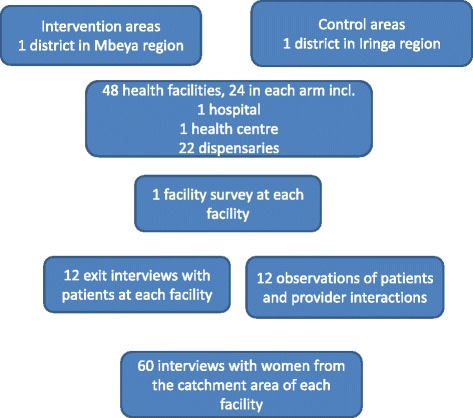
Overview of impact evaluation data collection tools and sample sizes

The impact evaluation relies on four tools that will be administered before the scheme is implemented and 17 months after implementation started: a health facility survey, a survey of patients exiting facilities, a client-provider interaction observation checklist, and a household survey of women who delivered in the previous 12 months (Fig. 2). The facility survey, exit interviews and client-provider observations will be conducted at 48 sampled facilities across intervention and comparison sites. The household survey will be administered to 3000 households within the catchment areas of these facilities to complement the data compiled during the facility survey [11].

### Data collection tools

The health facility survey aims to measure the effect of the KfW scheme on service availability and quality at the sampled facilities. The survey captures information on basic service provision within the facility (e.g. staffing levels, opening hours, facility management, as well as facility infrastructure), equipment and drug availability, and service utilization from patient registers and facility expenditures and revenues. The health facility survey will be administered to the facility in-charge or, in his or her absence, to a knowledgeable health worker or administrator. It also includes data extraction from patient registers and facility records.

The exit interview primarily intends to measure the effect of the KfW scheme on patient experience of care and the cost of services. Respondents eligible for interview include women of reproductive age (aged between 16 to 49 years) attending antenatal or postnatal care or childhood immunisation services within three months after delivery. A medical doctor or nurse will be trained to observe consultations with these patients and to complete a checklist to assess clinical care in relation to the national clinical guidelines.

A survey of women who had delivered within the previous 12 months will also be carried out. The women’s survey addresses the effects of the KfW scheme on care seeking and associated costs incurred during pregnancy and the postpartum as well as service satisfaction. The household head is also interviewed to ascertain health care utilisation rates and out of pocket payments for care seeking in the past month (outpatient care) and the past year (inpatient care) along with household socioeconomic status. The core indicators for each of the surveys are shown in Table 1.

**Table 1:** Overview of core indicators for impact evaluation

Survey type	Indicators	Data source
Service Utilisation	Average utilisation rates for outpatient care Average utilisation rates for inpatient care	Household survey
	% women delivering in a health facility	
	% of women who had any ANC	
	% of women who had 4 or more ANC visits	
	Average months pregnant at first ANC visit	
	% c-section rate	
	% newborn immunised before going home	
	% women who received postnatal care within 3 months of birth in a health facility	
	Number of PNC visits in a health facility within 3 months of birth	
	Average number of days after birth for first PNC visit	
	% of children fully immunised for polio (among appropriate age group)	
	% of children fully immunised for diphtheria, pertussis, and tetanus (DPT). (among appropriate age group)	
	% measles fully immunised for measles (among appropriate age group)	
	% women currently using a family planning method	
	Mean annual outpatient visits under 5	Health facility survey
	Mean annual outpatient visits all age groups	
	Mean annual inpatient admissions under 5	
	Mean annual inpatient admissions all age groups	
	Mean annual ANC service utilisation (all ANC and first ANC)	
	Mean annual delivery service utilisation (normal delivery)	
	Mean annual family planning visits	
	Mean number of under 1 year olds receiving DPT vaccine	
	Mean number of under 1 year olds receiving polio vaccine	
	Mean number of under 1 year olds receiving measles vaccine	
	Mean annual number of low birth weight babies	
	Mean annual c-sections	
	Mean annual number of stillbirths	
Quality of care	% patients prescribed drugs outside the facility	Household survey
	% babies weighed at birth	
	Average waiting time in minutes	Exit interview/observations
	Average consultation time in mins	
	% reporting overall satisfaction with quality	
	% did blood test during ANC	
	% took blood pressure during ANC	
	% prescribed iron tablets during ANC	
	% prescribed drugs for malaria during ANC	
	% counselling for HIV	
	% tested for HIV	
	% women examined during PNC	
	% babies weighed during PNC	
	Mean no. of clinical cadre	Health facility survey
	Mean no. of nursing cadre	
	Mean no. of paramedical cadre	
	% facilities offering 24 h delivery services	
	% facilities where skilled providers attend home deliveries	
	Average no of beds in the maternity ward for health centres/hospitals	
	% facilities with stock out of DPT vaccine type in past 90 days	
	% facilities with stock out of measles vaccine in past 90 days	
	% facilities with oxytocin stock outs in past 90 days	
	% facilities with oral rehydration salts stock outs in past 90 days	
	% facilities with stock outs of all anti-retrovirals in past 90 days	
	% facilities with partograph stock outs in past 90 days	
	% facilities reporting all contraceptive pill types stock out in past 90 days	
	% facilities reporting delivery kits stock out in past 90 days	
	% facilities reporting broken equipment disrupted the provision of services in past 90 days	
Financial protection	% patients paying for services	Household survey/observations
	% individuals who are members of CHF	Household survey
	% facilities with CHF	Facility survey
	% eligible women with NHIF card (intervention only)	Household survey
	Average out of pocket payments for outpatient care	
	Average out of pocket payments for inpatient care	
	% paying for delivery at public facility	
Equity	Average out of pocket payments for outpatient care (ratio of poor to least poor)	Household survey/observations
	Average out of pocket payments for inpatient care (ratio of poor to least poor)	
	Average utilisation rates for outpatient care (ratio of poor to least poor)	
	Average utilisation rates for inpatient care (ratio of poor to least poor)	
Health	Average weight of baby in kg	Household survey
	% breastfeeding within 1 h of birth	

### Sampling

The health facility is the primary sampling unit. Facilities were sampled from all facilities accredited by the NHIF within the selected districts. The government hospital and the health centre in each district were automatically selected (Fig. 2). A random sample of 22 dispensaries out of those which offered reproductive and child health (RCH) services were selected from each district. The total number of facilities sampled was 24 per district, representing over 60 % of all facilities in each of the two districts. The aim of the sampling procedure for the health facility survey was to seek district representation, therefore, no sample size calculation was carried out.

A total of 12 exit interviews and client provider observations will be carried out per facility at each round of data collection. The aim will be to achieve a balance between antenatal care (ANC) and postnatal care (PNC) or immunization service users within three months after birth (aiming for 6 ANC clients and 6 PNC or immunization clients per facility). Patients will be approached upon entry to the health facility regarding their participation in the exit interview. A series of screening questions will be used to identify eligible respondents who will be asked for their consent to participate in the study. Consenting respondents will be monitored from their time of arrival at the facility until their time of departure, and the waiting and consultation time will be measured using a stop watch. Patients and providers will also be asked for their consent for a medically trained interviewer to observe the consultation and complete an observation check list for ANC and PNC clients. Upon leaving the consultation room, the patient will then be asked for their consent to participate in the exit interview in a quiet location within the facility, at distance from providers and from other patients. At baseline, the criteria for selection will be that patients are uninsured. At endline the criteria for selection is that patients do not have any supplementary private health insurance, but patients with a CHF card or an NHIF card obtained through the KfW scheme will be eligible for interview.

For the household survey, the sample size calculation was based on the formula by Hayes and Bennett, 1999, adjusted for the cluster design of the study at the facility level [23]. We estimated that the required sample size to detect an 11 percentage point difference-in-differences increase in institutional deliveries (from 50 to 61 %), with an assumed coefficient of variation (standard deviation/mean) of the true rates between clusters within each group k value of 0.25, 90 % power, significance at 0.05 (two tailed test), and a 90 % response rate, was 60 households per cluster, equivalent to 1500 recently delivered women per study arm per round of data collection. Hence, the target sample was a total of 3000 recently delivered women per round of data collection. In order to identify eligible households, villages are sampled from the facility catchment area. Three villages will be sampled from the ward where the facility is located. All hamlets (comprising approximately 100 households) within the sampled villages will be identified and a random sample of four hamlets will be sampled. Five households will be sampled from each of the hamlets, amounting to a total of 60 households within each facility’s catchment area; households will be selected at random from the selected hamlets using a modified Expanded Programme of Immunisation (EPI) type sampling scheme that ensures an equal chance of any household being selected. In the sampled hamlet, the supervisor will aim to identify on average 3 households that scored “poor” and 2 that scored “nonpoor” (e.g. “average” or “rich”).

In order to be eligible for interview, households must include a woman who has delivered within the previous 12 months. At baseline the selection of households was also limited to those who were uninsured. At endline, eligible households included those who were uninsured, were insured with the CHF or insured by the NHIF through the KfW scheme. If there is an eligible woman, the supervisor will then ask permission from the respondent to complete a form to assess the socio economic status of the household. The supervisor will score the household from 1 to 3 on questions related to household characteristics (e.g. type of roof, water source, toilet facilities, average number of meals eaten per day, daily income, number of children in the house etc.). In the sampled hamlet, the supervisor will aim to identify on average 3 households that scored “poor” and 2 that scored “average” or “rich”. The objective will be to interview 40 households who are of poor or average wealth and 20 households who are not (least poor) per facility. The score sheet is the same tool originally proposed by the NHIF to identify beneficiaries.

### Process evaluation

The process evaluation will undertake ongoing descriptive and mixed methods assessment of the process of implementation, documenting the role and perspectives of key stakeholders at each stage of the process, and at each level of the health system, to ascertain how the scheme is implemented in practice. The evaluation will also track the degree to which implementation has occurred according to the design documentation (fidelity of implementation). Care will be taken to identify and monitor structural and contextual factors that may influence the observed implementation and outcomes. Ultimately, through implementation research we aim to determine what is the “core” of the intervention – the essential elements always necessary for it to be effective – and what is the “adaptive periphery” – i.e., those aspects of the intervention that can (and must) be adapted to fit the context.

The process evaluation will undertake three rounds of data collection at baseline, 14 and 18 months after implementation began in the selected intervention district (Mbarali). Focus group discussions and in-depth interviews will be carried out among a purposive sample of consenting individuals at different levels of the health system (community, facility, district, regional and national) to assess perspectives and attitudes towards the intervention as well as to routinely identify process bottlenecks. Three intervention facilities in Mbarali have been selected for an in-depth case study: a government hospital, health centre and dispensary. Staff at each of these health facilities will be interviewed, along with village leaders and focus group discussions with scheme beneficiaries and others in the community. These data will be triangulated with indicators for monitoring the fidelity of the intervention and its implementation process. The quantitative measures on content, coverage, frequency and duration will be derived from the household and exit surveys and project and health facility statistics. The analysis will also include a review of relevant project and policy documents and materials.

### Economic evaluation

The economic evaluation will assess the incremental cost-effectiveness of the KfW scheme relative to current practice. The study will be carried out from a societal perspective, which includes all agencies or bodies that are involved in implementation or who incur costs or may be affected by the intervention, for example: the implementers and the beneficiaries.

We will estimate the start-up and ongoing financial costs of the KfW scheme (i.e. all financial transactions made by the funder), as well as the economic costs, which values all resources required to set up and implement the scheme. Under economic costing, donated or subsidised items will be valued at market prices.

Project costs will be measured with reference to project accounts and through interviews with key implementation stakeholders at national, regional and district levels. Potential health system costs resulting from increased service use by scheme beneficiaries will be assessed by measuring any observed changes in staffing levels and bed numbers. Household costs and care seeking will be captured during the baseline and endline household surveys. Effectiveness is defined in relation to service coverage measured in the household survey.

A series of one way sensitivity analyses will be conducted to explore the impact of uncertainty on incremental cost-effectiveness.

### Data management

Household and exit interview data will be collected using hand held devices (Huawei IDEOS phones and Samsung Galaxy Tablets 7.0) loaded with Pendragon data collection software with skip and quality check functions to minimize data entry error. Facility survey data and client provider observations will be captured on paper and double entered into a pre-designed database. Data will be transferred into a Microsoft Access Database, and converted to Stata for analysis. Hard copies of questionnaires will be stored in a locked room. Electronic output will be de-identified.

Interviews and focus groups conducted as part of the process and economic evaluations will be conducted in Kiswahili by trained research assistants and recorded using audio digital recorders. Audio files will be transcribed by research assistants who conducted the interviews and will be translated into English by the bilingual researcher who also conducts the interviews. All transcripts will be imported into QSR Nvivo 8 for data management, for the process evaluation, and entered into Microsoft Excel for the economic evaluation.

### Analysis – quantitative data

Consistency checks will be conducted on the data from the baseline and endline surveys along with data cleaning. A comparison of all variables between intervention and control arms will be made at baseline through tests of differences in means using the Adjusted Wald F-test.

Socioeconomic status (SES) indices will be derived from data collected on household size and characteristics, access to utilities, durable asset ownership, food security, household expenditures, head of household marital status, highest level of education attained, and main occupation, using principal component analysis (PCA). We will rank individuals according to their index score and generate wealth terciles, three equally sized groups. Patient satisfaction data derived from a 3-point. Likert scale (e.g., dissatisfied = 1, neither satisfied nor dissatisfied = 2, satisfied = 3) will be analyzed by calculating mean scores for each variable.

At endline, we will compare the main outcome indicators for each of the survey tools between intervention and control arms, using data for 17 months of intervention implementation.

A difference-in-differences regression analysis will be conducted to assess the independent effect of the KfW scheme on outcomes controlling for all other individual, household and facility level factors which may influence the given outcome. The ordinary least squares linear regression model will be used and we will control for facility fixed effects. For household, exit and patient observations data, we will calculate robust standard errors, clustered at the facility level, to correct for correlation of the error terms across patients within facilities, and across households in facility catchment areas.

### Analysis– qualitative data

Qualitative data will be coded based on themes identified in the conceptual framework and adapted through an iterative process based on the data. Axial (line-by-line) coding will be conducted using Nvivo 8 software (QSR International Pty Ltd, Australia). A sample of the transcripts will be coded by a second researcher to insure reliability of the coding scheme. To validate findings, we will triangulate data across respondents and across methods.

### Ethical issues

The evaluation study was approved by the Institutional Review Board of the Ifakara Health Institute, the Tanzanian National Institute for Medical Research and the Population Council (P484). Letters were sent to District Executive Directors (DEDs) copied to District Medical Officers (DMOs) informing them of the study and its objectives prior to commencing the study. Prior to each round of data collection, calls were made to the DMOs to agree on dates for data collection. An information sheet was left at the DMO's office. Information sheets and consent forms were provided to all those participating in the study including patients, providers and households. Written consent was obtained prior to undertaking all in-depth interviews and focus group discussions conducted as part of the process evaluation.

## Discussion

The introduction of the KfW scheme in Tanzania aims to increase service utilization among poor pregnant women and their families, and also to stimulate better quality care for maternal and child health services. By promoting health insurance, the scheme also aims to sustain enrolment in community health insurance beyond the life time of the programme. However, the extent to which health insurance will effectively scale to reach those in need while avoiding adverse selection, and how health workers will respond to the scheme is as yet unclear.

This evaluation will contribute robust evidence on the impact and cost-effectiveness of a demand side financing programme of subsidized health insurance for the poor in a low-income setting, and shed light on the implementation process and challenges at different levels of the health system.

Previous studies have reported positive effects of health insurance on maternal health care use (e.g. [43, 44] and outcomes [45]. This study will provide a comprehensive assessment of the population and facility level impact of subsidized health insurance among poor pregnant women and their households in Tanzania, adding to a limited existing evidence base in the African region [29, 38]. This will add to our understanding of the impact of demand side financing schemes, and address innovative questions such as cost-effectiveness and equity effects. The study will also closely scrutinize the implementation process to assess implementation fidelity and status.

However, the evaluation will be conducted in only two districts, which may limit the generalisability of the findings to other regions. The process evaluation will ascertain the extent to which there is indeed variation in implementation across districts. A further limitation is the short time frame for the impact evaluation that evaluates effects over a 17 month period. A risk is that implementation has not yet been fully achieved which would limit the impact of the scheme. Again the process evaluation will shed light on the extent to which this is a factor.

## Abbreviations

ANC: antenatal care
CHF: Community health fund
DED: District executive director
DMO: District medical officer
EPI: Expanded programme of immunisation
FGD: Focus group discussion
MDG: Millennium development goal
NHIF: National Health Insurance Fund
PCA: Principal component analysis
PNC: postnatal care
RCH: Reproductive and child health
SES: Socio-economic status

## Footnotes

1 Selected facilities must have been in operation for at least 3 years, and have adequate infrastructure, equipment and staff, as ascertained by the NHIF.

2 Individual targeting is based on 8 components relating to: housing characteristics (housing materials, water and cooking fuel sources, and sanitation facilities); household remoteness from health providers; income; food security and the number of dependents including those with disabilities. Each component is scored between 1 and 3 depending on the degree to which the household is deemed to be poor (from 1 poorest, to 3 least poor), and then aggregated without weighting to obtain a total poverty score.
